# A MicroRNA Cluster in the *DLK1-DIO3* Imprinted Region on Chromosome 14q32.2 Is Dysregulated in Metastatic Hepatoblastomas

**DOI:** 10.3389/fonc.2020.513601

**Published:** 2020-11-12

**Authors:** Shohei Honda, Aniruddha Chatterjee, Anna L. Leichter, Hisayuki Miyagi, Masashi Minato, Sunao Fujiyoshi, Momoko Ara, Norihiko Kitagawa, Mio Tanaka, Yukichi Tanaka, Masato Shinkai, Kanako C. Hatanaka, Akinobu Taketomi, Michael R. Eccles

**Affiliations:** ^1^ Department of Gastroenterological Surgery I, Hokkaido University Graduate School of Medicine, Sapporo, Japan; ^2^ Department of Pathology, Dunedin School of Medicine, University of Otago, Dunedin, New Zealand; ^3^ Department of Surgery, Kanagawa Children’s Medical Center, Yokohama, Japan; ^4^ Department of Pathology, Kanagawa Children’s Medical Center, Yokohama, Japan; ^5^ Department of Surgical Pathology, Hokkaido University Hospital, Sapporo, Japan

**Keywords:** epigenetics, small nucleolar RNAs, microRNAs, hepatoblastoma, 14q32-encoded imprinted cluster genes

## Abstract

Hepatoblastoma (HB) is the most common malignant liver neoplasm in children. Despite progress in HB therapy, outcomes for patients with metastatic disease remain poor. Dysregulation of miRNA expression is one of the potential epigenetic mechanisms associated with pathogenesis of HB. However, miRNA profiles related to the different stages of HB tissues and cells, in particular of lung metastatic tumor cells, are unknown. In the present study, using array-based miRNA expression and DNA methylation analysis on formalin-fixed paraffin-embedded tissues, we aimed to identify miRNA changes that can discriminate between lung metastatic tumors, primary tumors (fetal and embryonal subtypes), and nontumorous surrounding livers. Our analysis demonstrated that a large cluster of microRNAs and snoRNAs located within the 14q32.2 *DLK1*-*DIO3* region showed a strikingly upregulated expression pattern in HB tumors, especially metastatic tumors, compared to normal liver tissues. This revealed dysregulation of miRNAs similar to that seen in a malignant stem-like subtype of hepatocellular carcinoma associated with poor prognosis. These findings in HB mirror similar findings made in multiple other cancer types. With further analysis this may in future allow stratification of different stages and types of HB tumors based on their miRNA profiles, which could lead to new approaches to diagnosis and treatment in progressive HB patients.

## Introduction

Hepatoblastoma (HB) is the most common malignant neoplasm of the liver in children. Despite progress in the therapy of HB, the outcome of patients with metastatic disease remains poor (1, 2). Approximately one fifth of HB patients have pulmonary metastasis at diagnosis, and recurrence of HB most frequently occurs in the lung. Among those patients who had residual lung disease after induction chemotherapy, but were able to undergo complete resection of the liver tumor, the overall survival rates in those whose lung tumors were completely resected, and of those whose lung tumors were incompletely resected, were 63.6% and 41.8%, respectively (3). To improve the prognosis, identification of novel molecular-genetic markers predictive of treatment outcome, and innovations that would allow personalized treatment are needed.

It is well known that mutations in beta-catenin are a hallmark of HB (4, 5). In contrast, results from recent whole-exome sequencing analyses showed other mutations are rarely seen in HB (3.9 - 4.6 mutations per tumor) (6, 7). The paucity of mutations has been reported in many pediatric tumors and may be correlated with their early age of onset (8). This paucity of mutations might also suggest that epigenetic aberrations are an important mechanism involved in the pathogenesis of HB.

Small non-coding RNAs, including microRNA (miRNA) and small nucleolar RNA (snoRNA), have been the focus of many studies in the last few decades and their fundamental role in cancer is currently well established. MiRNAs are a class of 19-25nt single-stranded RNA molecules that serve as major regulators of gene expression through their ability to bind to and post-transcriptionally inhibit the expression of specific target messenger RNAs. SnoRNAs are a class of 60-300 nt non-coding RNAs implicated in the chemical modification of ribosomal RNA, and their role in cellular regulation as well as a role in cancer development and progression has been highlighted (9).

We previously reported that aberrant DNA methylation of some tumor suppressor genes was related to poor outcome in HB patients and that disruption of imprinting status was implicated in its pathogenesis (10–12). Previous work on miRNA alterations in HB has shown that clustering of some miRNA expression profiles were related to several clinical aspects, including prognosis (7, 13–15). Therefore, dysregulation of epigenetic mechanisms, particularly miRNA expression and DNA methylation, could be a useful parameter for diagnosis as well as classification of HB. Primary liver cancers such as HB or hepatocellular carcinoma (HCC) often comprise heterogeneous types of cells with different histological features. HB tumors are classified as wholly epithelial, or of mixed epithelial and mesenchymal types. In the wholly epithelial type, there are two major subtypes, called the fetal subtype and the mixed fetal and embryonal subtype. Cairo et al. identified a 16-gene signature discriminating tumors with a fairly well-differentiated histology and a favorable prognosis against advanced and poorly differentiated tumors with a dismal outcome (16). Their signature defines HB prognostic subtypes that reflect liver developmental stage, which is in line with the ability of the miR signature to stratify patients with HB (13). A better understanding of the difference in molecular profiles related to the heterogeneity can aid in exploring molecular mechanisms of carcinogenesis and therapeutic options. Moreover, comparative molecular analyses between primary and metastatic tumors are useful to identify important molecules and pathways related to tumor progression. Therefore in the present study, we evaluated miRNA expression profiles related to different tissues and cell types in HB by using formalin-fixed, paraffin-embedded (FFPE) specimens. We previously confirmed that high quality data can be generated in HB FFPE sample analysis through miRNA array assays (17). The present study has identified remarkable dysregulation of microRNAs and snoRNAs located within the 14q32.2 *DLK1*-*DIO3* imprinted region, especially in metastatic HB tissues. The pattern of the miRNA dysregulation observed was typical of that seen in a malignant stem-like subtype of HCC associated with poor prognosis.

## Materials and Methods

### Patients

FFPE specimens were obtained from 16 patients referred to Hokkaido University Hospital and Kanagawa Children Medical Center for surgical treatment in between 2000 and 2016. Ten patients were male and six were female. Fourteen patients with a median age of 3.9 (0-10.5) years underwent primary tumor resection. Nine patients had lung metastasis (7, synchronous; 2, metachronous), and the nine metastatic tumor samples were resected at a median age of 7.6 (2.8–10.9) years (**Table 1**). The ethics committee at our institution approved the study protocol. For 6 of the patients, informed signed consent was provided by the parents. For the remaining 10 patients, the institutional ethics committee approved a web-based opt-out consent process, whereby each patient’s parents had the option of withdrawing the patient’s tissues and clinical information from the study.

**Table 1:** Patients’ characteristics and miRNA expression levels occurring within the indicated signaling pathways, calculated as logarithmically transformed probe intensity.

Case No.	Age (Y)	Sex	Lung metastasis	Sample type	Hippo signaling pathway			Myc signaling pathway							Wnt signaling pathway				C1/C2	*CHIC poor risk	
					mir-186	miR-125a-5p	miR-206	mir-371a	mir-371b	mir-372	mir-373	mir-100	let-7a-2	mir-125b-1	miR-4510	let-7i-3p	miR-624-5p	miR-885-5p	Cairo S, et al.	Age (> 8y)	Metastasis(present)
					(18)	(19)	(20)	(13)	(13)	(13)	(13)	(13)	(13)	(13)	(14)	(21)	(21)	(21)	(16)		
1	0	Female	–	N	1	10.92	1.3	0.67	1.29	0.65	0.91	1.2	0.63	0.59	0.88	1.21	0.98	10.28	C1	No	No
	0			F	0.69	10.82	0.89	0.81	1.13	1.19	0.65	0.72	0.8	0.77	0.46	0.92	0.58	7.37			
2	0.9	Female	–	N	0.38	10.18	0.69	0.74	1.31	0.69	1.04	0.42	0.81	0.73	0.67	1.07	0.89	10.25	C1	No	No
	0.9			F	0.55	11.46	0.63	1.15	1.23	0.8	0.67	1	0.96	0.69	1.14	1.51	0.45	7.51			
	0.9			E	1.08	10.05	1.21	1.36	1.45	0.8	0.77	0.9	0.67	0.87	0.61	0.44	0.85	6.22			
3	1.1	Female	–	N	1.77	1.41	1.09	0.62	1.94	0.75	0.96	1.38	0.92	0.17	0.77	1.18	0.8	1.79	C1	No	No
	1.1			F	0.93	8.76	0.73	1.34	1.58	0.87	1.44	1.1	0.65	1.41	1.13	0.52	0.71	6.01			
4	1.3	Male	meta	N	1.43	6.47	1.13	1.13	1.52	1.29	1.44	0.83	0.93	1.24	0.99	0.92	1.18	8.48	C1	No	Yes
	1.3			E	1.52	7.56	3.8	1.32	1.43	1.09	1.13	0.42	0.54	1.18	2.11	0.62	0.88	1.89			
	4.9			M	1.23	8.29	0.78	1.31	1.81	1.05	1.28	0.71	0.86	0.96	1.31	0.48	0.9	1.47			
5	1.4	Male	sync	N	0.86	9.16	0.87	1.64	1.39	1.24	1.35	0.91	0.81	0.51	1.06	0.63	0.52	9.9	C2	No	Yes
	1.4			F	1.35	10.39	1.14	1.26	1.38	0.81	2.45	0.74	0.89	1.36	0.63	1.54	0.96	5.56			
	1.4			E	1.92	10.2	0.82	1.37	1.06	0.8	1.53	0.28	1.08	0.94	1.84	1.98	1.5	6.72			
6	1.8	Male	–	N	0.65	9.8	0.93	0.7	2.25	0.75	1.08	0.59	0.96	0.9	1.22	1.05	0.46	10.7	C2	No	No
	1.8			F	1.04	11.79	4.98	0.96	1.42	0.92	0.44	0.78	0.82	0.89	1.7	1.01	0.64	8.46			
	1.8			E	1.02	10.86	1.36	1.93	1.93	1.31	0.25	0.75	0.85	0.34	1.28	1.28	0.51	6.77			
7	2.3	Female	–	F(biopsy)	0.86	10.91	1.06	0.81	1.41	1.33	0.93	0.43	1.41	0.69	0.97	1.04	0.45	8.29	C1	No	No
	2.5			N	1.3	10.18	1.39	0.74	0.89	0.86	0.48	0.8	1.25	0.31	1.6	1.03	0.34	9.6			
	2.5			F	0.72	9.72	1.4	1.21	1.81	0.96	0.63	0.39	0.86	0.77	1.09	1.08	0.52	6.38			
8	2.7	Male	sync	N	0.93	9.02	0.99	0.97	1.7	1.04	0.59	0.77	0.62	0.58	1.04	1.8	1.27	9.81	C1	No	Yes
	2.7			F	1.04	10.9	0.76	0.89	1.2	0.6	0.93	1.61	0.91	1.68	1.65	2.47	1.11	8.92			
	2.7			E	1.4	10.76	0.78	1.08	1	1.15	1	0.51	0.96	1.6	1.87	0.99	1.18	8.23			
	2.8			M	1.11	10.38	1.56	1.71	1.59	0.75	0.8	0.6	0.98	0.46	1.11	1.06	0.98	6.89			
9	3.6	Male	–	N	1.49	5.42	1.23	1.87	1.17	0.68	2.48	1.06	0.73	1.51	0.63	1.22	0.91	0.91	C1	No	No
	3.6			F	0.9	9.62	0.71	0.87	1.47	0.94	1.16	1.24	1.27	1.62	1.67	1.11	1.22	7.18			
	3.6			E	1.06	8.58	0.92	1.76	1.12	0.92	1.78	1.18	0.6	0.58	0.73	0.57	0.84	5.08			
10	6.3	Male	sync	N	0.64	8.9	1.35	1.51	1.44	0.83	2.2	0.64	0.66	1.33	1.01	1.11	0.72	9.08	C1	No	Yes
	6.0			M	1.15	9.59	1.05	1.1	1.03	0.82	0.67	1.01	0.88	0.78	0.52	1.48	1.13	7.8			
11	6.2	Female	–	N	1	6.76	0.83	0.75	1.35	1.34	1.14	0.72	1.65	0.79	0.71	0.79	1.65	1.34	C1	No	No
	6.2			F	0.96	9.06	1.29	1.62	1.6	1.06	1.1	0.74	1.15	1.92	0.64	0.26	0.7	3.37			
	6.2			E	1.13	7.89	0.79	0.79	1.47	1.39	0.41	0.76	0.59	2.41	0.38	0.7	1.05	3.37			
12	7.4	Female	sync	N	1.77	8.1	0.93	1.07	2.36	0.61	1.14	0.41	0.66	1.17	0.95	0.91	1.25	7.29	C1	No	Yes
	7.4			F	1.01	9.62	1.03	1.33	1.56	0.63	1.01	0.59	0.82	0.61	0.74	0.51	1.19	7.13			
	7.4			E	1.06	7.38	0.68	2.06	2.04	0.9	0.83	0.85	0.95	1.04	0.7	0.51	0.91	4.2			
	7.6			M	1.36	8.83	1.23	1.08	1.75	0.73	1.01	0.7	0.62	0.77	1.53	0.86	1.06	6.18			
13	7.7	Male	meta	M	1.93	11.52	4.61	1.9	1.5	1.3	0.58	0.86	0.74	1.67	1.75	1.3	0.72	5.81	C1	No	Yes
	8.1			M	0.67	10.84	1.87	1.26	1.56	0.63	0.48	0.87	0.75	0.56	0.45	0.7	0.77	6.35			
14	9.3	Male	sync	N	0.75	7.3	0.95	0.75	1.61	0.47	1.04	0.32	0.78	1.44	1.46	1.35	0.95	9.69	C2	Yes	Yes
	9.3			E	1.08	9.45	1.32	1.42	1.29	1.33	0.76	1.23	0.73	1.08	1.03	1.49	1.15	7.3			
	9.8			M	0.65	11.32	2.68	2.29	1.3	5.07	3.53	0.62	0.64	0.95	1.26	0.9	0.45	2.95			
15	10.5	Male	sync	N	0.74	9.65	0.65	0.86	1.49	0.61	0.69	0.8	0.37	1.76	0.55	0.83	0.89	4.84	C2	Yes	Yes
	10.5			E	0.88	8.68	0.85	2.08	1.72	0.87	1.3	0.25	0.64	2.21	1.13	0.49	1.21	3.89			
	10.8			M	1.19	7.29	1.63	0.88	1.81	0.98	0.7	0.47	1.18	0.26	1.08	0.83	0.85	6.48			
16	10.9	Male	sync	M	0.84	10.72	1.58	1.21	1.31	1.06	0.48	0.7	1.1	0.9	1.58	0.79	0.52	8.49	NA	Yes	Yes

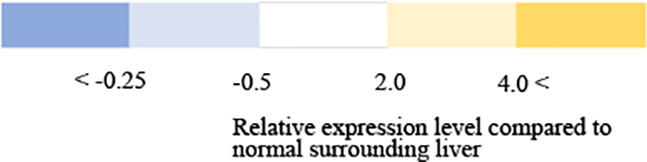

The Log2 difference between tumor and normal liver indicates the expression relative to normal liver tissue, and is colored in each cell as depicted by the fold-difference color key at the bottom, where the darker colors represent greater differences in expression levels. Metastatic tumor (M), embryonal subtype (E), fetal subtype (F), and normal surrounding liver (N).

Age, patients’ age when each tumor resection was performed; meta, metachronous; sync, synchronous; [number] represents reference no.

M, metastatic tumor; E, embryonal subtype; F, fetal subtype; N, normal surrounding liver.

*CHIC, Children’s Hepatic tumors International Collaboration.

### RNA Extraction and miRNA Array

The FFPE specimens included tumor cells from 9 metastatic tumors, 21 primary tumors (11 fetal subtypes and 10 embryonal subtypes), and normal cells from 14 nontumorous surrounding liver samples. After dissecting the specimens macroscopically under a light microscope with a sterile scalpel, ensuring tissues were collected well away from tissue boundaries and stored systematically, which enabled us to avoid contamination by normal tissues or mesenchymal components, we extracted 44 RNA samples using a miRNeasy FFPE Kit (Qiagen, Hilden, Germany) according to the manufacturer’s instructions. We conducted a quality check (QC) of the RNA samples measuring the concentration of total RNA and OD 260/280 ratio using DS-11 NanoPad (DeNovix, Wilmington, USA) or NanoDrop ND-2000 (Thermo Fisher Scientific, Waltham, USA), and RNA integrity number (RIN) using Agilent 2100 Bioanalyzer using Agilent RNA 6000 Nano Kit (Agilent Technologies, Santa Clara, USA). All the data for QC is shown in **Supplementary Table 1**. The OD 260/280 ratios for all the samples ranged between 1.58 and 1.95; however, RIN numbers were relatively low at between 1.3 and 3.2. The miRNA expression was profiled using GeneChip^®^ miRNA4.0 arrays following the manufacturer’s recommended protocols.

### DNA Extraction and Methylation Array

We extracted DNA samples from the tissue FFPE samples mentioned above. To extract the DNA, we used a QIAamp DNA FFPE Tissue Kit (Qiagen) according to the manufacturer’s instructions. We conducted a quality check of the DNA samples using RT-PCR, following the Infinium HD FFPE QC Assay Protocol, and we confirmed that 12 samples from six nontumorous surrounding liver (8N, 9N, 10N, 11N, 12N, and 14N in **Table 1**) and six metastatic tumor (4M, 8M, 10M, 12M, 14M, and 15M in **Table 1**) were appropriate for the methylation assay. We next conducted genome-wide methylation analyses using an Infinium HumanMethylation450 BeadChip (Illumina) and the 12 DNA samples, following the Illumina Infinium HD Methylation protocol. This array includes 485,577 cytosine positions in the human genome (482,421 CpG sites (99.4%), 3,091 non-CpG sites and 65 random SNPs). We linked the UCSC Genome Browser annotation (version hg19 of the human reference genome) to each of the CpG sites on the array. Based on the UCSC chromosome annotation, we then screened for probes that were located in the *DLK1-DIO3* imprinted region.

### Data Analysis and Statistical Analysis of miRNA Data

A workflow describing the study design, which involved the miRNA and snoRNA microarray data analysis is shown in **Supplementary Figure 1**. All raw data were normalized by the use of a tool that uses Robust Multichip Analysis (RAM) plus detection above background (DABG) algorithms and analyzed using Transcriptome Analysis Console ver. 4.0 (Affymetrix, Santa Clara, CA).

Statistical analysis and data visualization of heatmap with hierarchical clustering were performed by Qlucore Omics Explorer (QOE) (Qlucore AB, Lund, Sweden). The differential expression of miRNAs in the different tissue cell types was analyzed using multi-group comparisons with ANOVA (false discovery rate (FDR) < 0.05). Variance filtering was used to reduce the noise and the filtering threshold was set of 0.52 applied by QOE. To compare differentially expressed miRNAs between normal surrounding liver tissues and primary or metastatic tumors, a two-group comparison (with > 2 fold mean expression change and one-way ANOVA, FDR < 0.05) was used. Data visualization of box plots was performed using GraphPad Prism software (version 6, La Jolla, CA) and *P*-values were calculated by Tukey’s test. The average Euclidean distance was adopted for hierarchical clustering. To perform pathway enrichment analysis and targets prediction, we used Diana mirPath v.3 web-based computational tool (http://microrna.gr/mirpath). We used the “pathways union” option of the miRPath software. *P*-values were obtained by the Fisher’s exact test as enrichment analysis method and the FDR was estimated using the Benjamini and Hochberg method. All pathways showing *P*-values < 0.05 were considered significantly enriched between the groups under comparison (22). To represent the HB risk stratification as described by Cairo et al. (2010), we classified the HB tumors into C1/C2 subclasses that are related to Cm1/Cm2 subclasses determined by miR expression profiles (13). In **Table 1**, C2 tumors were defined as tumors which showed mir-371 and/or 373 upregulation, compared to the matched nontumorous surrounding livers.

## Results

### Identification of Differentially Expressed MiRNAs in Four Different Tissue Types in HB

**Figure 1:**
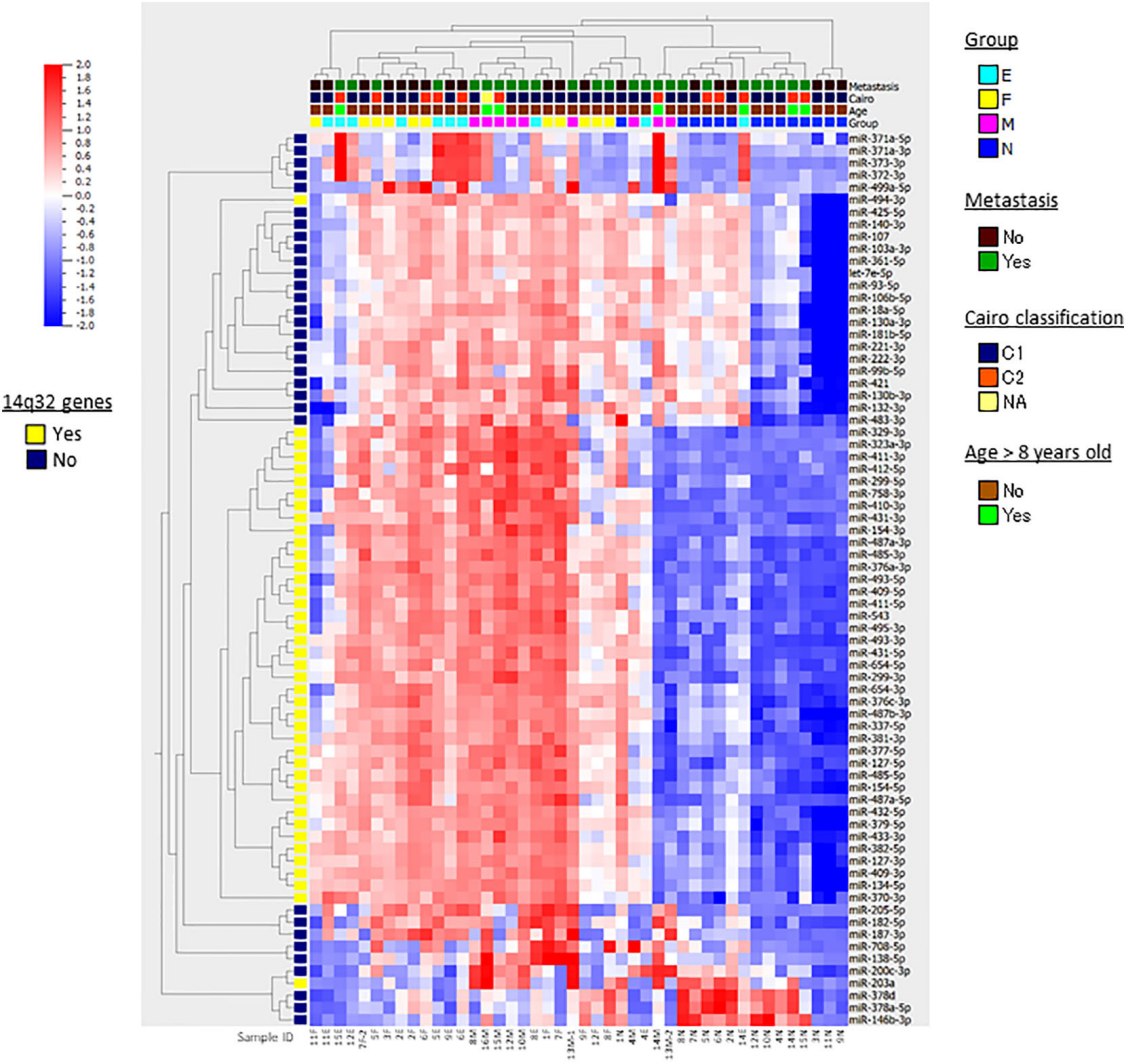
Heatmap and hierarchical clustering. The results of hierarchical clustering of 73 differentially expressed miRNAs in multi-group comparisons with ANOVA in 44 HB samples are presented in the heatmap diagram. Each row represents a miRNA and each column represents a sample. The colored boxes shown at the top of the heat map illustrate Group, the stages and tissue cell types of the samples (N, nontumorous surrounding liver; F, fetal subtype; E, embryonal subtype; M, metastatic tumor); Metastasis, presense of lung metastasis at diagnosis; Cairo classification, C1/C2 classification based on the expression of Myc-related miRNAs; Age, age > 8 years old or not. The colored box shown at the left of the heat map illustrates the genes located at 14q32. Colors range from red (high expression) to blue (low expression). At the bottom, the sample IDs are shown.

Following the strategy outlined in the workflow diagram (**Supplementary Figure 1**), we compared miRNA expression levels in four different tissue and cell types in HB: nontumorous liver tissue surrounding HB (n=14), primary HB tumor tissues of two subtypes [fetal (n=11) and embryonal (n=10)], and metastatic tumor tissue (n=9), and detected 73 differentially expressed miRNAs in multi-group comparisons using ANOVA (FDR < 0.05). The genomic locations, fold changes, and statistical values of the miRNAs are shown in **Table 2**. The results of hierarchical clustering of the expression of these 73 miRNAs in the 44 tissue samples are presented in the heatmap diagram (**Figure 1**), showing that, with a few exceptions, almost all the nontumorous surrounding liver samples expressed most of the identified miRNAs at relatively low levels, and that these 73 miRNAs became upregulated in primary and metastatic tumors. The exceptions included one nontumorous surrounding liver sample that expressed most of the miRNAs at relatively high levels, and one embryonal tumor plus two metastatic tumors that expressed a subgroup of the miRNAs at relatively low levels, while five nontumorous surrounding liver samples expressed a smaller but different subgroup of the miRNAs at relatively high levels. Hierarchical clustering showed that the metastatic tumors, and the fetal and embryonal subtypes of primary tumors did not segregate from each other. Overall, these results suggest that by hierarchical clustering the identified differentially expressed miRNAs were able to discriminate between normal surrounding liver tissue and HB tumor tissue (whether it be primary or metastatic), but that clustering of the miRNA expression was unable to discriminate between metastatic tumors and the primary tumors of the two subtypes (**Figure 1**).

**Table 2:** 73 differentially expressed miRNAs in four different types of cells.

miR Name	Accession	*p*-value	FDR	Chromosome	*Genomic Position	**Fold Change		
						M	E	F
miR-181b-5p	MIMAT0000257	0.000313875	0.000992871	chr1	198828054-198828076	9.5	5.9	8.9
**miR-205-5p**	MIMAT0000266	0.001991707	0.005416047	chr1	209605511-209605532	66.3	109.8	32.7
miR-425-5p	MIMAT0003393	0.005762251	0.014641785	chr3	49057632-49057654	2.8	1.6	2.2
miR-378d	MIMAT0018926	0.010992384	0.025815448	chr4	5925006-5925025	-10.1	-13.6	-6.1
miR-378a-5p	MIMAT0000731	0.009306511	0.022440539	chr5	149112392-149112413	-8.2	-9.3	-5.1
miR-93-5p	MIMAT0000093	0.016715075	0.037011952	chr7	99691438-99691460	2.3	1.6	1.2
miR-182-5p	MIMAT0000259	0.000675694	0.002014089	chr7	129410287-129410310	23.0	12.4	18.7
miR-106b-5p	MIMAT0000680	0.000673778	0.002014089	chr7	99691666-99691686	3.4	1.9	1.7
miR-107	MIMAT0000104	0.02029133	0.043084332	chr10	91352513-91352535	1.6	1.3	2.3
miR-146b-3p	MIMAT0004766	0.001277737	0.003600896	chr10	104196313-104196334	-10.6	-12.2	-8.5
miR-130a-3p	MIMAT0000425	0.013705112	0.031239593	chr11	57408725-57408746	3.8	2.6	3.4
miR-483-3p	MIMAT0002173	0.016537205	0.037011952	chr11	2155372-2155392	11.8	8.8	10.8
miR-708-5p	MIMAT0004926	0.006560128	0.01640032	chr11	79113121-79113143	14.4	2.1	6.9
miR-200c-3p	MIMAT0000617	1.02295E-06	8.34512E-06	chr12	7072905-7072927	26.9	-4.1	-2.1
miR-18a-5p	MIMAT0000072	0.000136605	0.000504136	chr13	92003010-92003032	8.0	5.0	5.0
miR-203a	MIMAT0000264	0.000274513	0.000924988	chr14	104583806-104583827	-4.1	-6.4	4.2
miR-127-5p	MIMAT0004604	3.28566E-07	4.04884E-06	chr14	101349338-101349359	28.2	13.5	15.8
miR-127-3p	MIMAT0000446	2.25408E-06	1.20476E-05	chr14	101349372-101349393	29.6	12.7	17.5
miR-134-5p	MIMAT0000447	1.31517E-06	8.86308E-06	chr14	101521031-101521052	20.2	14.4	12.6
miR-154-5p	MIMAT0000452	5.66419E-07	5.79746E-06	chr14	101526106-101526127	22.1	11.9	19.2
miR-154-3p	MIMAT0000453	2.02166E-06	1.16058E-05	chr14	101526142-101526163	12.7	14.8	22.8
**miR-299-5p**	MIMAT0002890	0.00030853	0.000992871	chr14	101490137-101490158	32.0	8.7	19.7
miR-299-3p	MIMAT0000687	1.00761E-07	2.39227E-06	chr14	101490169-101490190	20.4	13.4	14.6
**miR-376c-3p**	MIMAT0000720	1.44147E-07	2.39227E-06	chr14	101506069-101506089	48.0	22.9	59.0
miR-370-3p	MIMAT0000722	1.4522E-07	2.39227E-06	chr14	101377523-101377544	15.7	19.9	9.8
**miR-376a-3p**	MIMAT0000729	1.64805E-08	1.27724E-06	chr14	101506455-101506475	69.3	36.9	82.1
miR-377-5p	MIMAT0004689	1.27936E-06	8.86308E-06	chr14	101528393-101528414	28.7	10.2	16.6
**miR-379-5p**	MIMAT0000733	1.49029E-06	9.23978E-06	chr14	101488408-101488428	49.8	26.7	32.3
miR-381-3p	MIMAT0000736	1.17417E-06	8.86308E-06	chr14	101512305-101512326	21.8	20.0	28.3
**miR-382-5p**	MIMAT0000737	5.8365E-08	2.26164E-06	chr14	101520653-101520674	33.0	29.0	24.0
miR-337-5p	MIMAT0004695	6.42573E-06	3.01815E-05	chr14	101340852-101340872	30.7	18.4	27.9
**miR-323a-3p**	MIMAT0000755	0.000227554	0.000783797	chr14	101492119-101492139	33.6	12.6	8.8
**miR-431-5p**	MIMAT0001625	4.09569E-08	2.11611E-06	chr14	101347363-101347383	257.0	146.8	146.6
miR-431-3p	MIMAT0004757	3.65702E-07	4.04884E-06	chr14	101347406-101347427	27.5	16.7	13.0
**miR-433-3p**	MIMAT0001627	1.16987E-07	2.39227E-06	chr14	101348286-101348307	72.2	45.6	32.8
miR-329-3p	MIMAT0001629	5.8346E-05	0.000220576	chr14	101493171-101493192	17.5	10.8	11.9
**miR-409-5p**	MIMAT0001638	1.73525E-06	1.03448E-05	chr14	101531651-101531673	64.7	36.1	49.3
miR-409-3p	MIMAT0001639	2.54168E-07	3.58146E-06	chr14	101531683-101531704	25.5	22.7	19.4
miR-412-5p	MIMAT0026557	0.000189664	0.000668135	chr14	101531802-101531824	19.8	13.1	11.0
miR-410-3p	MIMAT0002171	2.1092E-05	8.83583E-05	chr14	101532298-101532318	18.7	11.2	8.4
miR-485-5p	MIMAT0002175	1.38906E-06	8.97103E-06	chr14	101521764-101521785	28.3	14.3	19.4
**miR-485-3p**	MIMAT0002176	3.05593E-06	1.5789E-05	chr14	101521801-101521822	59.4	19.9	41.0
**miR-487a-5p**	MIMAT0026559	1.87669E-05	8.0802E-05	chr14	101518795-101518816	35.6	16.9	19.8
**miR-487a-3p**	MIMAT0002178	7.03521E-07	6.0581E-06	chr14	101518831-101518852	147.1	74.5	94.8
**miR-493-5p**	MIMAT0002813	5.68274E-06	2.75258E-05	chr14	101335412-101335433	43.9	21.6	33.4
**miR-493-3p**	MIMAT0003161	8.29291E-09	1.27724E-06	chr14	101335453-101335474	193.9	99.4	93.0
**miR-432-5p**	MIMAT0002814	1.5434E-07	2.39227E-06	chr14	101350833-101350855	118.7	80.2	59.1
miR-494-3p	MIMAT0002816	0.007463012	0.018361378	chr14	101496018-101496039	3.5	3.1	3.8
miR-495-3p	MIMAT0002817	1.40687E-07	2.39227E-06	chr14	101500141-101500162	31.9	16.6	22.8
**miR-487b-3p**	MIMAT0003180	4.76728E-06	2.38364E-05	chr14	101512842-101512863	83.6	47.8	56.4
**miR-411-5p**	MIMAT0003329	2.12687E-06	1.17738E-05	chr14	101489677-101489697	61.0	21.3	46.1
miR-411-3p	MIMAT0004813	0.000287224	0.000947228	chr14	101489712-101489733	19.6	6.6	12.3
miR-654-5p	MIMAT0003330	3.62352E-07	4.04884E-06	chr14	101506571-101506592	30.6	18.2	21.5
**miR-654-3p**	MIMAT0004814	1.29197E-05	5.72159E-05	chr14	101506606-101506627	49.7	16.5	43.9
**miR-758-3p**	MIMAT0003879	6.55547E-07	5.97705E-06	chr14	101492408-101492429	41.8	22.3	12.4
miR-543	MIMAT0004954	5.98448E-07	5.79746E-06	chr14	101498370-101498391	27.0	13.4	26.4
miR-138-5p	MIMAT0000430	0.001449068	0.004010814	chr16	56892439-56892461	10.8	1.2	1.9
miR-140-3p	MIMAT0004597	0.009410549	0.022440539	chr16	69967045-69967065	2.1	1.8	2.5
miR-132-3p	MIMAT0000426	0.013017126	0.030114247	chr17	1953223-1953244	7.3	5.2	6.1
miR-187-3p	MIMAT0000262	0.002625966	0.007017668	chr18	33484798-33484819	14.5	14.5	11.3
let-7e-5p	MIMAT0000066	0.00501861	0.012964742	chr19	52196046-52196067	2.7	2.2	3.2
miR-99b-5p	MIMAT0000689	0.000969498	0.002782817	chr19	52195871-52195892	3.7	1.9	3.2
miR-371a-5p	MIMAT0004687	4.75396E-05	0.000184216	chr19	54290934-54290953	3.9	17.8	1.9
miR-371a-3p	MIMAT0000723	0.000185971	0.000668135	chr19	54290970-54290992	2.4	18.5	1.2
miR-372-3p	MIMAT0000724	2.16787E-05	8.84261E-05	chr19	54291185-54291207	16.4	98.1	1.3
**miR-373-3p**	MIMAT0000726	1.29903E-06	8.86308E-06	chr19	54292002-54292024	33.9	142.4	4.4
miR-103a-3p	MIMAT0000101	0.0193977	0.041758938	chr20	3898188-3898210	1.7	1.5	2.3
miR-499a-5p	MIMAT0002870	0.018524962	0.040441818	chr20	33578211-33578231	1.9	2.7	1.6
miR-130b-3p	MIMAT0000691	0.000415097	0.001286799	chr22	22007643-22007664	6.3	5.3	3.3
miR-221-3p	MIMAT0000278	4.67814E-05	0.000184216	chrX	45605608-45605630	5.2	3.1	6.5
miR-222-3p	MIMAT0000279	1.11958E-05	5.10397E-05	chrX	45606442-45606462	9.6	5.7	9.9
miR-361-5p	MIMAT0000703	0.004202336	0.011040036	chrX	85158686-85158707	3.2	1.9	3.0
miR-421	MIMAT0003339	0.000727357	0.002127177	chrX	73438227-73438249	10.1	4.9	5.9

FDR, false discovery rate; **miRNAs** in bold type, top 21 upregulated miRNAs analyzed with DIANA-miRPath v.3; miRNAs with underline, four downregulated miRNAs analyzed with DIANA-miRPath v.3.; *Genomic position are based on GRCh37/hg19 build.; **Fold changes were calculated by weighted average comparing M (metastatic tumors), E (embryonal subtypes) and F (fetal subtypes) to nontumorous surrounding liver samples using Transcriptome Analysis Console ver. 4.0.

In addition to hierarchical clustering, we compared the miRNA expression profiles of nontumorous surrounding liver vs. primary and metastatic tumors in a two-group comparison (with > 2-fold mean expression change and one-way ANOVA, FDR < 0.05), and identified 60, 51, and 81 differentially expressed miRNAs in the fetal subtype, embryonal subtype and metastatic tumor tissue compared to normal tissue, respectively (**Supplementary Figure 2**). However, except for several miRNAs that were expressed at relatively low levels, we ultimately were unable to identify single miRNAs using this approach that were significantly differentially expressed in metastatic tumors versus either fetal or embryonal subtype tumor.

### Pathway Enrichment Analysis and Target Prediction

**Figure 2:**
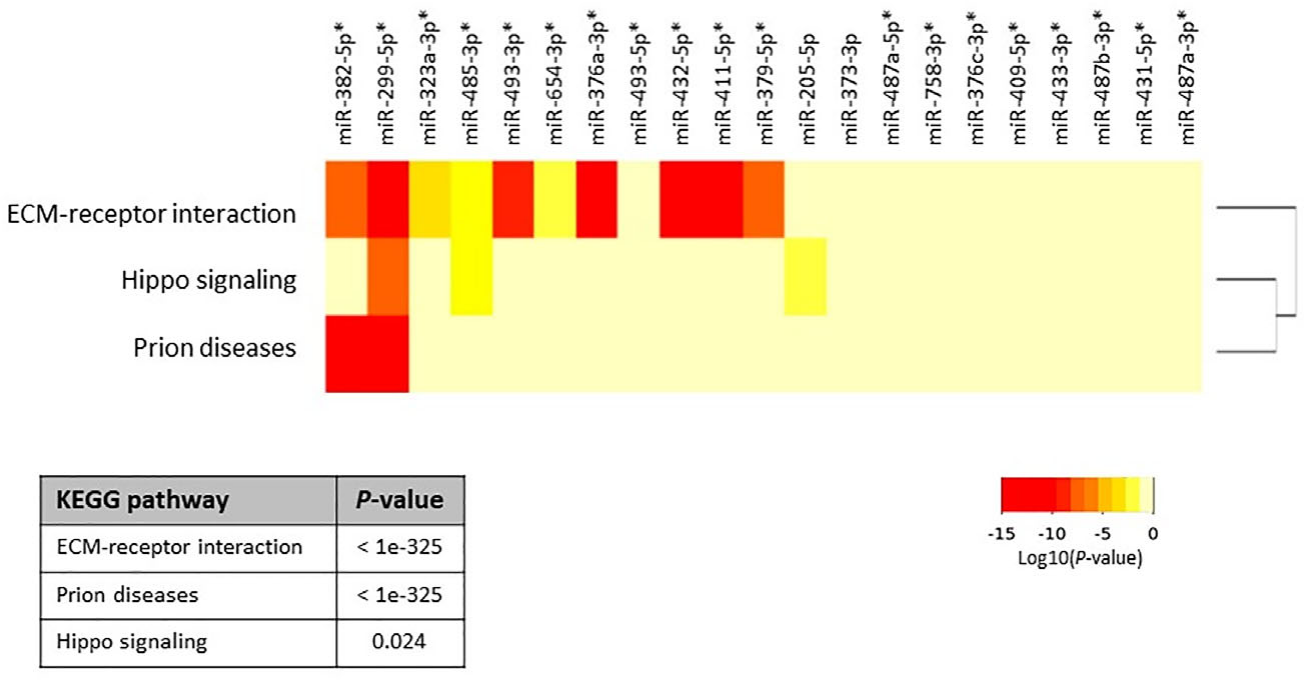
Heatmap of upregulated miRNAs versus significantly enriched functional pathways. In the heatmap, darker colors represent higher statistical significance of the Log10(*P-*value) as indicated by the color key at the bottom. Pathways showing *P*-values < 0.05 were considered significantly enriched between the groups under comparison. The attached dendrogram on the Y-axis depicts hierarchical clustering results for the pathways. Asterisks show the miRNAs that are located within the *DLK1*-*DIO3* region on chromosome 14q32.2. The figure was developed from the output of Diana mirPath v.3.

Next, we aimed to investigate whether specific pathways involving up- and down-regulated miRNAs were altered in primary and metastatic HB tumors. For this analysis, we calculated the enrichment of multiple miRNA target genes and compared each set of miRNA targets to all known Kyoto Encyclopedia of Genes and Genomes pathways to identify altered pathways. We first investigated whether co-expression of the top 21 upregulated miRNAs (highlighted as bold in **Table 2**, and selected on the basis of fold-difference in expression) in metastatic tumors compared to those in the normal surrounding liver tissue could affect some signaling pathways. The miRNA versus pathway heatmap showed that signaling pathways regulating Hippo (*P*-value, 0.024), also known as the Salvador/Warts/Hippo pathway, which controls organ size in animals (5), were significantly enriched in the case of the upregulated miRNAs (**Figure 2**). Moreover, when we investigated all the upregulated genes in **Table 2** in the pathway enrichment analysis, 18 KEGG pathways were significantly enriched (**Supplementary Table 2**), which included Hippo (*P*-value, 8.28e-10), TGF-beta (*P*-value, 0.0073), and p53 (*P*-value, 6.02e-05) signaling pathways. On the other hand, when we examined the targets of the four downregulated miRNAs, miR-378d, miR-378a-5p, miR-146b-3p and miR-203a, in metastatic tumors versus normal surrounding tissue for enriched functional pathways, this yielded biosynthesis of unsaturated fatty acids (hsa01040) at the top of the list (*P*-value, 0.033).

### DLK1-DIO3 Genomic Imprinted miRNA Cluster at 14q32.2

**Figure 3:**
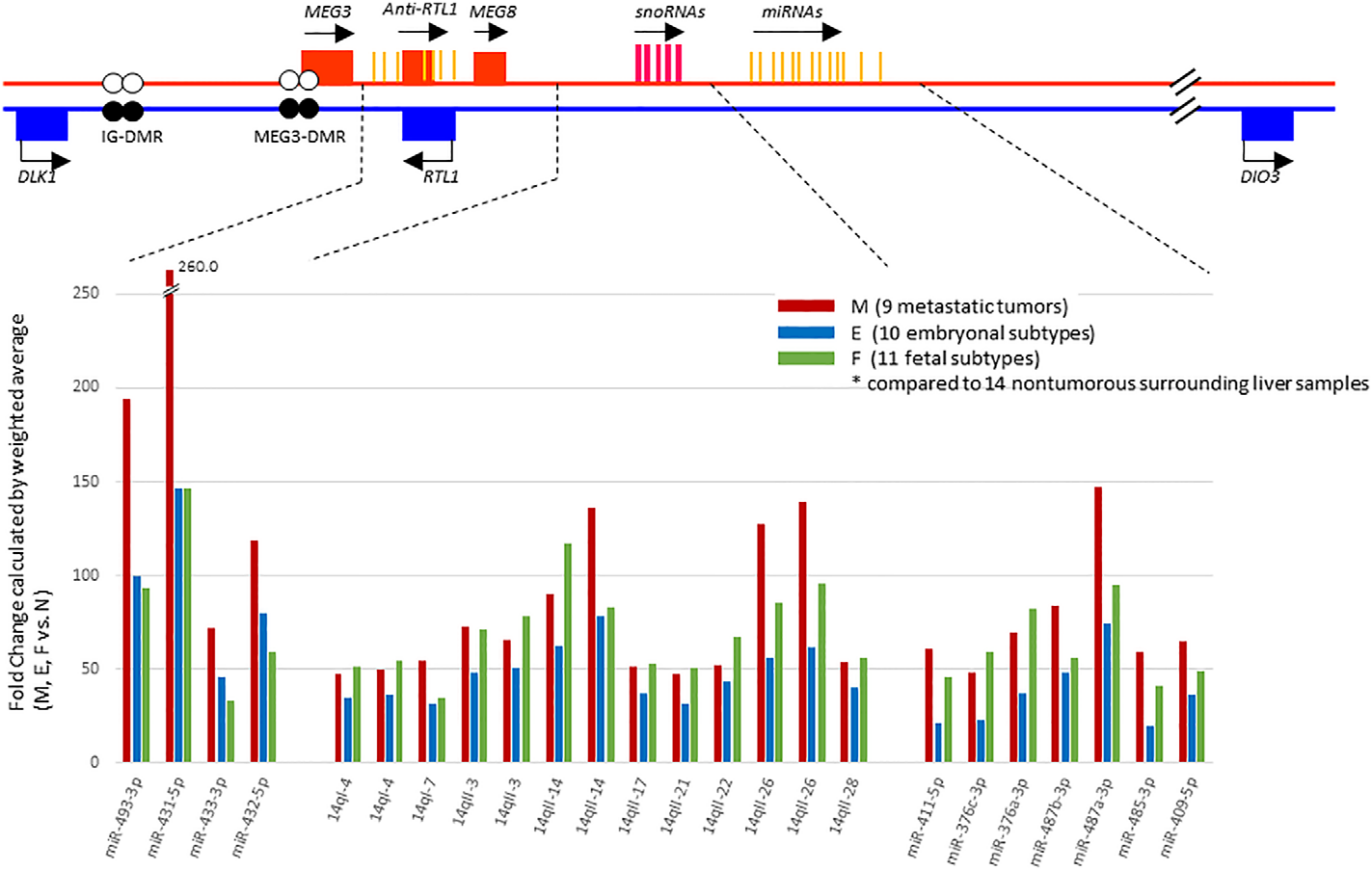
Schema of the *DLK1-DIO3* imprinted locus on chromosome 14q32.2, and miRNAs and small nucleolar RNAs (snoRNAs) located in the region. The *DLK1-DIO3* locus consists of three known protein-coding genes including *DLK1*, *RTL1*, *DIO3*, noncoding RNAs including at least three lncRNAs, and numerous snoRNAs and miRNAs. The maternal chromosome is in red and the paternal chromosome in blue. Differentially methylated regions, called IG-DMR and MEG3-DMR, are shown as a pair of circles (filled circles, methylated; open circles, unmethylated). On the lower panel, representation of expression levels of the most strongly upregulated miRNAs and snoRNAs shown in fold change calculated by weighted average comparing metastatic tumor (M), embryonal subtype € and fetal subtype (F) to normal surrounding liver samples(N) using Transcriptome Analysis Console ver. 4.0.

In a previous study (17), we had noted that chromosome 14 contained the highest percentage (~15%) of the total complement of microRNAs expressed in HBs upon mapping of miRNAs to each chromosome, and that these miRNAs were concentrated in a relatively small region of chromosome 14, the *DLK1-DIO3* imprinted region, which contains a miRNA cluster at 14q32.2. We therefore examined the chromosomal locations of the differentially expressed miRNAs in our tumor samples to identify whether this might reveal miRNA clusters enriched in a specific genomic location. Surprisingly, we found 55% (40 out of the 73) of all the identified miRNAs were located within the *DLK1*-*DIO3* region on chromosome 14q32.2 (**Table 2**). Moreover, 19 of the top 21 miRNAs investigated in **Figure 2** are located within the *DLK1*-*DIO3* region on chromosome 14q32.2. The miRNAs that cluster in the 14q32.2 region are separated by a cluster of putative C/D box small nucleolar RNAs, therefore, we evaluated the expression of 93 miRNAs and 54 small nucleolar RNAs (snoRNAs) located in this region in the tumor samples and compared that with the normal surrounding liver samples to identify patterns of differential expression. It showed that 54 of these miRNAs (58.0%) and 31 small nucleolar RNAs (57.4%) were upregulated with > 2-fold mean expression change in at least one type of tumor tissue. As shown in **Figure 3**, the increased miRNA and snoRNA expression levels, which were calculated by weighted average using Transcriptome Analysis Console ver. 4.0, were overall the highest in the metastatic tumors. This was most apparent in the snoRNAs, which were highly upregulated with greater than 50-fold mean expression change in at least one type of tumor tissue. Comparing mean fold differences in expression, differential expression of miRNAs and snoRNAs occurred at the *DLK1-DIO3* locus in HB metastatic and primary tissues versus normal liver tissues (**Supplementary Figure 3**).

To examine whether metastasis-associated changes in miRNA expression occurred, we used matched trios of metastatic, primary, and normal liver tissues derived from the same patients. Relative ratios of miRNA expression in the matched tissues were calculated in primary tumors (n=21) and metastatic tumors (n=6) divided by expression in the matched normal liver tissue. In this analysis, we excluded three metastatic tumor samples (21M, 23M-1, and 23M-2) because of lack of the matched normal samples. Comparison of the ratios showed that 23 miRNAs and 8 snoRNAs were significantly upregulated in the metastatic compared to primary tumor tissues (**Supplementary Table 3**, and **Supplementary Figure 4**).

To determine whether the miRNAs that were upregulated in this region had any specific function, we investigated all of the 40 identified upregulated miRNAs using the pathway enrichment analysis tool. This showed that only two KEGG pathways, Prion diseases (hsa05020) (*P*-value, <1e-325) and ECM-receptor interaction (hsa04512) (*P*-value, <1e-325), were significantly enriched, both of which were similarly yielded at the top of the list in the previous analyses (**Figure 2** and **Supplementary Table 1**).

**Figure 4:**
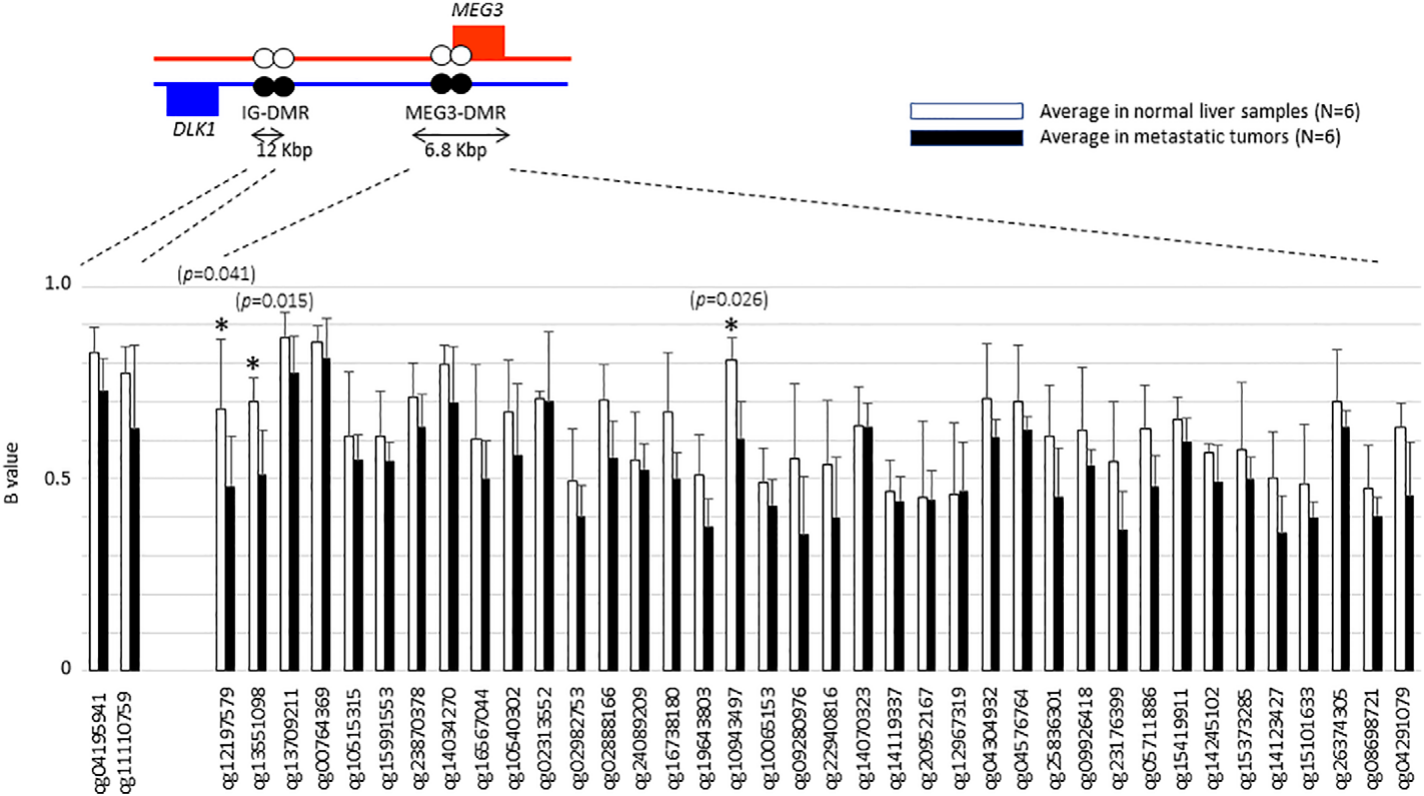
Histogram showing the average of β-values at each probe that are located in the *DLK1-DIO3* imprinted region to compare between metastatic tumors and normal liver tissues. Illumina TargetIDs are shown below the horizontal axis of the figure. Three probes with asterisk showed significant difference in methylation levels. *P* values were calculated by Mann-Whitney U analysis.

**Figure 5:**
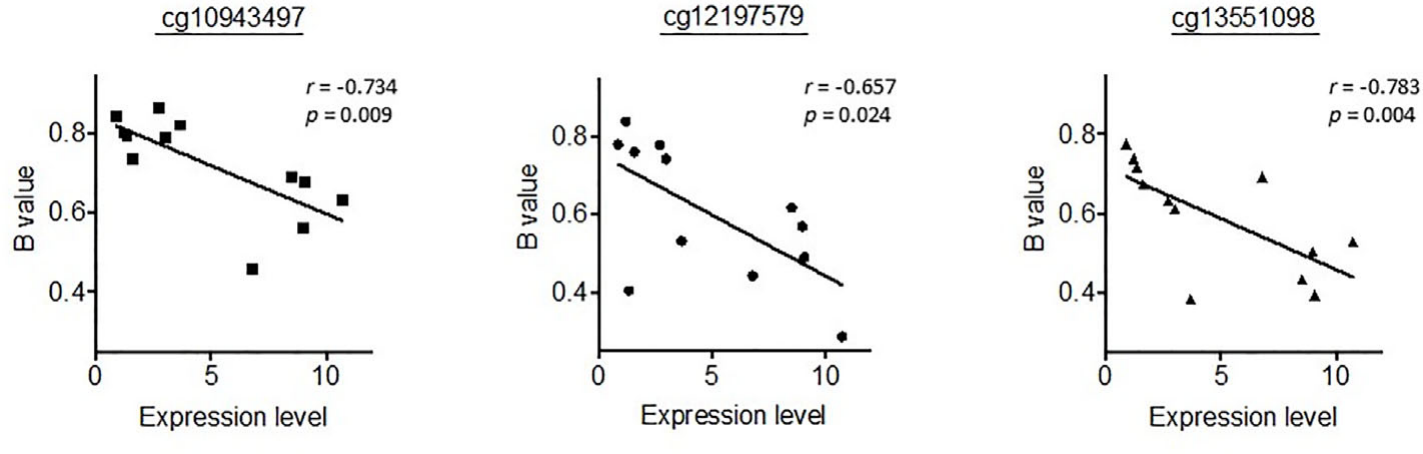
Representation of the correlation between the methylation levels at the three probes significantly differentiated in **Figure 4** and the expression levels of miR-433-3p, which had the highest correlation coefficient. *r*, Spearman’s rank correlation rho.

We then went on to further analyse whether differential methylation between metastatic tumor tissues and normal liver tissues occurred at two regulatory DMRs (Differentially Methylated Regions), which are responsible for controlling the imprinted expression of genes, including *MEG3*, in the *DLK1-DIO3* imprinted region. We observed that the methylated CpG dinucleotides in these two DMRs all displayed an average loss of DNA methylation when analysed in a series of six metastatic tumour tissues, as compared to DNA methylation levels in six normal liver tissues (**Figure 4**). Three CpG probes located in the *MEG3-*DMR showed significantly lower methylation levels in metastatic tumor tissues compared to normal liver tissues using Mann-Whitney U analysis. As shown in **Figure 5** and **Supplementary Table 4**, low methylation levels at the three probes were significantly correlated with the upregulation of the miRNAs and snoRNAs shown in **Figure 3**. While these results are not sufficient to demonstrate a causal association between hypomethylation and increased miRNA expression from the *DLK1-DIO3* imprinted region, they are nevertheless consistent with elevation of miRNA and snoRNA expression levels in the *DLK1-DIO3* imprinted region potentially resulting from an overall loss of DNA methylation in the locus. Also consistent with our results, Luk et al. (2011) had shown that miRNAs in the *DLK1-DIO3* imprinted region were overexpressed in a stem-like subtype of hepatocellular carcinoma (23), and very recently Carrillo-Reixach et al. (2020) have reported that genes in the *DLK1-DIO3* imprinted region are hypomethylated and overexpressed in HB, enabling stratification of patient outcome (24).

### Expression of miRNAs Participating in Key Signaling Pathways Involved in the Pathogenesis of HB Tumors

Several studies have explored the role of miRNAs in some key signaling pathways altered in the pathogenesis of HB tumors (14) and references therein. The known miRNAs participating in a key signaling pathway, the Hippo pathway, include miR-186, miR-125a, and miR-206 (14). We compared the expression levels of two oncogenic miRNAs (miR-125a and miR-206) and one tumor-suppressive miRNA (mir-186), by comparing the probe intensities in each tumor tissue type to that of normal surrounding liver tissue in each of the 14 cases where this comparison could be done (**Table 1**). As shown in the colored-cell patterns in **Table 1**, we found that miR-125a-5p was upregulated in 10 out of the 14 cases (71%) in at least one tumor tissue type compared to normal surrounding liver. In contrast, changes in the expression of miRNAs participating in the Wnt (miR-4510, let-7i-3p, miR-624-5p, and miR-885-5p), and Myc (miR-371 cluster and miR-100/let-7a-2/miR-125b-1 cluster) signaling pathways, which have also been highlighted in HB (5), were unremarkable, except miR-885-5p, which was downregulated in 10 and upregulated in 4 out of the 14 cases in at least one tumor tissue type compared to normal surrounding liver, respectively. In the present analysis, we found no apparent associations between any alterations in those miRNA expressions and risk stratification factors, such as C1/C2 classification, or age at diagnosis and the presence of metastasis (**Table 1**). On the other hand, when we compared the expression levels of 14q32 transcripts in the *DLK1-DIO3* imprinted region with these same prognostic factors in the 21 primary tumor samples, we identified seven miRNAs and two snoRNAs, which were significantly upregulated in the six primary tumors that were classified into the C2 classification (**Supplementary Figure 5**). Furthermore, in the seven primary tumors which had metachronous or synchronous metastasis, three miRNAs and one snoRNA were significantly upregulated (**Supplementary Figure 6**). There were no differentially upregulated 14q32 small RNAs when comparing age at diagnosis of more than versus less than 8 years of age.

## Discussion

HB tumors demonstrate a diverse range of histological phenotypes and clinical behaviors, which may arise as a result of the proliferation of transformed liver stem cells or of early hepatic precursors with varying degrees of differentiation (7). Furthermore, the presence of metastasis is the most important prognostic factor for HB patients (1–3). The use of FFPE specimens has enabled us to separately collect different types and stages of HB tumor tissues. Our analysis has demonstrated that HB shows distinct miRNA profiles related to both normal and HB tumor type and stage.

Our data provide the first description, to our knowledge, of miRNA profiles investigated in primary tumour and lung metastatic HB tumors. However, the main limitation of our data was that, due to HB being a very rare tumor, only a relatively small number of the clinical samples were examined. Nonetheless, miRNAs and snoRNAs located in the 14q32.2 cluster showed significantly upregulated expression in metastatic tumors. Interestingly, our results resemble recent findings within a stem-like subtype of HCC, which is known to be associated with overexpression of miRNAs located in this cluster (23), and they are also very similar to a recent report showing that genes in the *DLK1-DIO3* imprinted region are hypomethylated and overexpressed in HB, enabling stratification of patient outcomes (24). These studies suggest that certain molecular mechanisms, such as Wnt signaling pathways or TGF-beta, might be related to the tumorigenesis of this unique stem-like subtype of HCC. Indeed, in their very recent paper Carrillo-Reixach et al. (24) investigated a large cohort of HB patients, and demonstrated that overexpression and hypomethylation of genes in the 14q32 *DLK1-DIO3* imprinted region, which includes the largest known cluster of miRNAs and snoRNAs in the human genome, is associated with HB patient outcome, and activation of the Wnt/beta-catenin pathway. Activation of the Wnt/β-catenin is a hallmark in HB (4, 25), and the Hippo pathway has also been reported in HB (26, 27). Hippo signaling transcription factors YAP and TAZ are known to regulate the β-catenin destruction complex and to orchestrate Wnt responses (28, 29). These findings suggest that dysregulation of 14q32 clusters might play an important role in HB pathogenesis *via* interaction with Wnt/β-catenin/Hippo signaling pathways. The strong upregulation of imprinted genes in the 14q32 locus, *DLK1*, and *MEG3*, which are abundantly expressed in fetal liver, has also been shown in HB (16). Products of genes targeted by miRNAs from this locus are expected to regulate receptor tyrosine kinase-activated pathways in the pathogenesis of relapsing-remitting multiple sclerosis (30).

Several previous studies investigating copy-number variations in HB showed that copy-number alterations in the chromosomal region are rarely seen (7, 31), suggesting that it is unlikely that gene amplification was the cause of upregulation of the genes and miRNAs at this locus. This is also supported by our findings that reduced average methylation was identified at two differentially methylated regions (DMRs), which have previously been shown to control transcription from imprinted genes in the locus, including *MEG3*. In contrast to our findings, Oshima G, et al., showed that expression of 14q32-encoded miRNAs was a favorable prognostic factor in patients with metastatic colorectal cancer (32). Further investigations of aberrant methylation at imprinting control regions are warranted to determine whether disruption of the imprinting status of this locus is involved in the pathogenesis of HB. In addition, HCC patients with overexpressed 14q32.2 miRNAs exhibited shorter survival times (23), therefore, miRNAs from this locus might also become prognostic biomarkers and candidates for targeted therapies designed to treat HB patients with progressive disease. These miRNAs should therefore be further explored using more clinical specimens for their diagnostic and prognostic use in HB.

Among miRNAs that participate in key signaling pathways previously identified as being involved in the pathogenesis of HB tumors, miR-125a-5p might frequently function as an oncogene in the development of both primary and metastatic HB tumors. Suppression of miR-125a-5p upregulates the Tafazzin (TAZ)-EGFR signaling pathway, and has been shown to promote retinoblastoma proliferation. It has also been suggested to have a potential role in regulating the Hippo pathway (19). In summary, new findings involving miRNAs could serve to provide alternative therapeutic strategies for the treatment of HB, especially in patients with metastatic disease.

## Conclusion

Stratifying different types and stages of primary and metastatic HB tumors based on their miRNA profiles could lead to new approaches to diagnosis and treatment in HB patients with progressive disease. The present work has identified a surprisingly high proportion of upregulated miRNAs located within the 14q32.2 locus in HB tumors, and particularly in metastatic tumors. Discovering more about the upregulation of both miRNAs and snoRNAs on the 14q32.2 locus could improve our understanding towards innovating better therapeutic strategies to improve clinical outcomes of progressive HB, as well as potentially elucidating their role in stem-like subtypes of HCC.

## Data Availability Statement

The datasets presented in this study can be found in online repositories. The names of the repository/repositories and accession number(s) can be found below: the NCBI Gene Expression Omnibus (GSE153089).

## Ethics Statement

The studies involving human participants were reviewed and approved by Hokkaido University Hopital Ethics Committee. Approval code is 010-0202. Written informed consent to participate in this study was provided by the participants’ legal guardian/next of kin.

## Author Contributions

Conceptualization, SH, AC, and ME. Methodology, SH, AL, HM, MM, SF, and MA. Validation: SH, AC, AL, and ME. Investigation: SH, NK, MS, AC, and AT. Pathological analysis: MT, YT, and KH. Writing—original draft preparation: SH, SF, MA, and AT. Writing—review and editing: AC, AL, and ME. Project administration: AT and ME. Funding acquisition: SH, AT, and ME. All authors contributed to the article and approved the submitted version.

## Funding

This research was funded by JSPS KAKENHI Grant Numbers JP15K10915 and JP18K07781. ME and AC were funded by the New Zealand Institute for Cancer Research Trust. AC would like to thank Rutherford Discovery Fellowship program (Royal Society of New Zealand) for funding his position.

## Conflict of Interest

The authors declare that the research was conducted in the absence of any commercial or financial relationships that could be construed as a potential conflict of interest.

## Supplementary Material

Supplementary Figure 1 Workflow describing the study design.

Supplementary Figure 2 Venn diagram showing the relationship between miRNAs which were up-/down-regulated in different HB stages of tissue and cell types compared to nontumorous surrounding liver. Each stage and cell type is illustrated as N, nontumorous surrounding liver; F, fetal subtype; E, embryonal subtype; M, metastatic tumor.

Supplementary Figure 3 Differential expression of miRNAs and snoRNAs at the 14q32.2 imprinted locus in HB metastatic and primary tissues versus normal liver tissues.

Supplementary Figure 4 Comparison of the expressions of 14q32 small RNAs which showed significant difference in primary tumors (n=21) vs. metastatic tumors (n=6). In this analysis, three metastatic tumors (21M, 23M-1 and 23M-2) were excluded because there were no matched normal liver tissues to calculate the relative ratio. Ten of the 31 small RNAs shown in **Supplementary Table 3** are in this figure as representatives. The Y-axis shows the relative ratio of (Signal intensity in tumor/Signal intensity in the matched normal sample). The number above the boxes represents *p*-values calculated by Tukey’s test. The illustrative box plots present the median by a line in the box with the 10th, 90th percentile and the range of the relative ratios. P, primary tumors; M, metastatic tumors.

Supplementary Figure 5 Comparison of the expression level of 14q32 miRNAs and snoRNAs which showed significant differences in C2 (n=6) versus C1 tumors (n=15). The Y-axis shows the relative ratio of expression levels (signal intensity in tumor/signal intensity in the matched normal sample). The number above the boxes represents the *p*-values calculated by Tukey’s test. The box and whisker plots indicate the median as a line in the box, which demarcates the 10th, 90th percentiles of the range of the relative ratios.

Supplementary Figure 6 Comparison of the expression level of 14q32 miRNAs and snoRNAs which showed significant difference in tumors exhibiting metachronous or synchronous metastasis (n=7) versus tumors that did not (n=14). The Y-axis shows the relative ratio of expression levels (signal intensity in tumor/signal intensity in the matched normal sample). The number above the boxes represents the *p*-values calculated by Tukey’s test. The box and whisker plots indicate the median as a line in the box, which demarcates the 10th, 90th percentiles of the range of the relative ratios.

## References

[1] Hishiki T Matsunaga T Sasaki F Yano M Ida K Horie H Outcome of hepatoblastomas treated using the Japanese Study Group for Pediatric Liver Tumor (JPLT) protocol-2: report from the JPLT. Pediatr Surg Int (2011) 27(1):1–8. 10.1007/s00383-010-2708-0 20922397

[2] von Schweinitz D Hepatoblastoma: recent developments in research and treatment. Semin Pediatr Surg (2012) 21(1):21–30. 10.1053/j.sempedsurg.2011.10.011 22248967

[3] Hishiki T Watanabe K Ida K Hoshino K Iehara T Aoki Y The role of pulmonary metastasectomy for hepatoblastoma in children with metastasis at diagnosis: Results from the JPLT-2 study. J Pediatr Surg (2017) 52(12):2051–5. 10.1016/j.jpedsurg.2017.08.031 28927977

[4] Koch A Denkhaus D Albrecht S Leuschner I von Schweinitz D Pietsch T Childhood hepatoblastomas frequently carry a mutated degradation targeting box of the beta-catenin gene. Cancer Res (1999) 59(2):269–73. 9927029

[5] Leichter AL Sullivan MJ Eccles MR Chatterjee A MicroRNA expression patterns and signalling pathways in the development and progression of childhood solid tumours. Mol Cancer (2017) 16(1):15. 10.1186/s12943-017-0584-0 28103887 PMC5248531

[6] Jia D Dong R Jing Y Xu D Wang Q Chen L Exome sequencing of hepatoblastoma reveals novel mutations and cancer genes in the Wnt pathway and ubiquitin ligase complex. Hepatology (2014) 60(5):1686–96. 10.1002/hep.27243 24912477

[7] Sumazin P Chen Y Trevino LR Sarabia SF Hampton OA Patel K Genomic analysis of hepatoblastoma identifies distinct molecular and prognostic subgroups. Hepatology (2017) 65(1):104–21. 10.1002/hep.28888 27775819

[8] Vogelstein B Papadopoulos N Velculescu VE Zhou S Diaz LA Jr. Kinzler KW Cancer genome landscapes. Science (2013) 339(6127):1546–58. 10.1126/science.1235122 PMC3749880 23539594

[9] Romano G Veneziano D Acunzo M Croce CM Small non-coding RNA and cancer. Carcinogenesis (2017) 38(5):485–91. 10.1093/carcin/bgx026 PMC6248440 28449079

[10] Honda S Arai Y Haruta M Sasaki F Ohira M Yamaoka H Loss of imprinting of IGF2 correlates with hypermethylation of the H19 differentially methylated region in hepatoblastoma. Br J Cancer (2008) 99(11):1891–9. 10.1038/sj.bjc.6604754 PMC2600691 19034281

[11] Honda S Haruta M Sugawara W Sasaki F Ohira M Matsunaga T The methylation status of RASSF1A promoter predicts responsiveness to chemotherapy and eventual cure in hepatoblastoma patients. Int J Cancer (2008) 123(5):1117–25. 10.1002/ijc.23613 18537155

[12] Honda S Minato M Suzuki H Fujiyoshi M Miyagi H Haruta M Clinical prognostic value of DNA methylation in hepatoblastoma: Four novel tumor suppressor candidates. Cancer Sci (2016) 107(6):812–9. 10.1111/cas.12928 PMC4968605 26991471

[13] Cairo S Wang Y de Reynies A Duroure K Dahan J Redon MJ Stem cell-like micro-RNA signature driven by Myc in aggressive liver cancer. Proc Natl Acad Sci U.S.A. (2010) 107(47):20471–6. 10.1073/pnas.1009009107 PMC2996672 21059911

[14] Cristobal I Sanz-Alvarez M Luque M Carames C Rojo F Garcia-Foncillas J The Role of MicroRNAs in Hepatoblastoma Tumors. Cancers (Basel) (2019) 11(3):409. 10.3390/cancers11030409 PMC6468899 30909459

[15] Leichter AL Purcell RV Sullivan MJ Eccles MR Chatterjee A Multi-platform microRNA profiling of hepatoblastoma patients using formalin fixed paraffin embedded archival samples. Gigascience (2015) 4:54. 10.1186/s13742-015-0099-9 26613016 PMC4660849

[16] Cairo S Armengol C De Reynies A Wei Y Thomas E Renard CA Hepatic stem-like phenotype and interplay of Wnt/beta-catenin and Myc signaling in aggressive childhood liver cancer. Cancer Cell (2008) 14(6):471–84. 10.1016/j.ccr.2008.11.002 19061838

[17] Chatterjee A Leichter AL Fan V Tsai P Purcell RV Sullivan MJ A cross comparison of technologies for the detection of microRNAs in clinical FFPE samples of hepatoblastoma patients. Sci Rep (2015) 5:10438. 10.1038/srep13505 26039282 PMC4453922

[18] Ruan T He X Yu J Hang Z MicroRNA-186 targets Yes-associated protein 1 to inhibit Hippo signaling and tumorigenesis in hepatocellular carcinoma. Oncol Lett (2016) 11(4):2941–5. 10.3892/ol.2016.4312 PMC4812570 27073580

[19] Zhang Y Xue C Zhu X Zhu X Xian H Huang Z Suppression of microRNA-125a-5p upregulates the TAZ-EGFR signaling pathway and promotes retinoblastoma proliferation. Cell Signal (2016) 28(8):850–60. 10.1016/j.cellsig.2016.04.002 27094723

[20] Yang Y Del Re DP Nakano N Sciarretta S Zhai P Park J miR-206 Mediates YAP-Induced Cardiac Hypertrophy and Survival. Circ Res (2015) 117(10):891–904. 10.1161/CIRCRESAHA.115.306624 26333362 PMC4747867

[21] Indersie E Lesjean S Hooks KB Sagliocco F Ernault T Cairo S MicroRNA therapy inhibits hepatoblastoma growth in vivo by targeting β-catenin and Wnt signaling. Hepatol Commun (2017) 1(2):168–83. 10.1002/hep4.1029 PMC5721429 29404451

[22] Vlachos IS Zagganas K Paraskevopoulou MD Georgakilas G Karagkouni D Vergoulis T DIANA-miRPath v3.0: deciphering microRNA function with experimental support. Nucleic Acids Res (2015) 43(W1):W460–6. 10.1093/nar/gkv403 PMC4489228 25977294

[23] Luk JM Burchard J Zhang C Liu AM Wong KF Shek FH DLK1-DIO3 genomic imprinted microRNA cluster at 14q32.2 defines a stemlike subtype of hepatocellular carcinoma associated with poor survival. J Biol Chem (2011) 286(35):30706–13. 10.1074/jbc.M111.229831 PMC3162431 21737452

[24] Carrillo-Reixach J Torrens L Simon-Coma M Royo L Domingo-Sàbat M Abril-Fornaguera J Epigenetic footprint enables molecular risk stratification of hepatoblastoma with clinical implications. J Hepatol (2020) 73(2):328–41. 10.1016/j.jhep.2020.03.025 PMC12452110 32240714

[25] Wei Y Fabre M Branchereau S Gauthier F Perilongo G Buendia MA Activation of beta-catenin in epithelial and mesenchymal hepatoblastomas. Oncogene (2000) 19(4):498–504. 10.1038/sj.onc.1203356 10698519

[26] Tao J Calvisi DF Ranganathan S Cigliano A Zhou L Singh S Activation of β-catenin and Yap1 in human hepatoblastoma and induction of hepatocarcinogenesis in mice. Gastroenterology (2014) 147(3):690–701. 10.1053/j.gastro.2014.05.004 24837480 PMC4143445

[27] Min Q Molina L Li J Adebayo Michael AO Russell JO Preziosi ME β-Catenin and Yes-Associated Protein 1 Cooperate in Hepatoblastoma Pathogenesis. Am J Pathol (2019) 189(5):1091–104. 10.1016/j.ajpath.2019.02.002 Erratum in: Am J Pathol. 2019 Aug;189(8):1680. PMC6521893 30794807

[28] Azzolin L Zanconato F Bresolin S Forcato M Basso G Bicciato S Role of TAZ as mediator of Wnt signaling. Cell (2012) 151(7):1443–56. 10.1016/j.cell.2012.11.027 23245942

[29] Azzolin L Panciera T Soligo S Enzo E Bicciato S Dupont S YAP/TAZ incorporation in the β-catenin destruction complex orchestrates the Wnt response. Cell (2014) 158(1):157–70. 10.1016/j.cell.2014.06.013 24976009

[30] Baulina N Osmak G Kiselev I Popova E Boyko A Kulakova O MiRNAs from DLK1-DIO3 Imprinted Locus at 14q32 are Associated with Multiple Sclerosis: Gender-Specific Expression and Regulation of Receptor Tyrosine Kinases Signaling. Cells (2019) 8(2):130. 10.3390/cells8020133 PMC6406543 30743997

[31] Arai Y Honda S Haruta M Kasai F Fujiwara Y Ohshima J Genome-wide analysis of allelic imbalances reveals 4q deletions as a poor prognostic factor and MDM4 amplification at 1q32.1 in hepatoblastoma. Genes Chromosomes Cancer (2010) 49(7):596–609. 10.1002/gcc.20770 20461752

[32] Oshima G Poli EC Bolt MJ Chlenski A Forde M Jutzy JMS DNA Methylation Controls Metastasis-Suppressive 14q32-Encoded miRNAs. Cancer Res (2019) 79(3):650–62. 10.1158/0008-5472.CAN-18-0692 30538122

