# Supplementary figures and images for: A MicroRNA Cluster in the DLK1-DIO3 Imprinted Region on Chromosome 14q32.2 Is Dysregulated in Metastatic Hepatoblastomas
Source: Front Oncol. 2020 Nov 12;10:513601. doi: 10.3389/fonc.2020.513601 (PMC7689214; doi:10.3389/fonc.2020.513601)

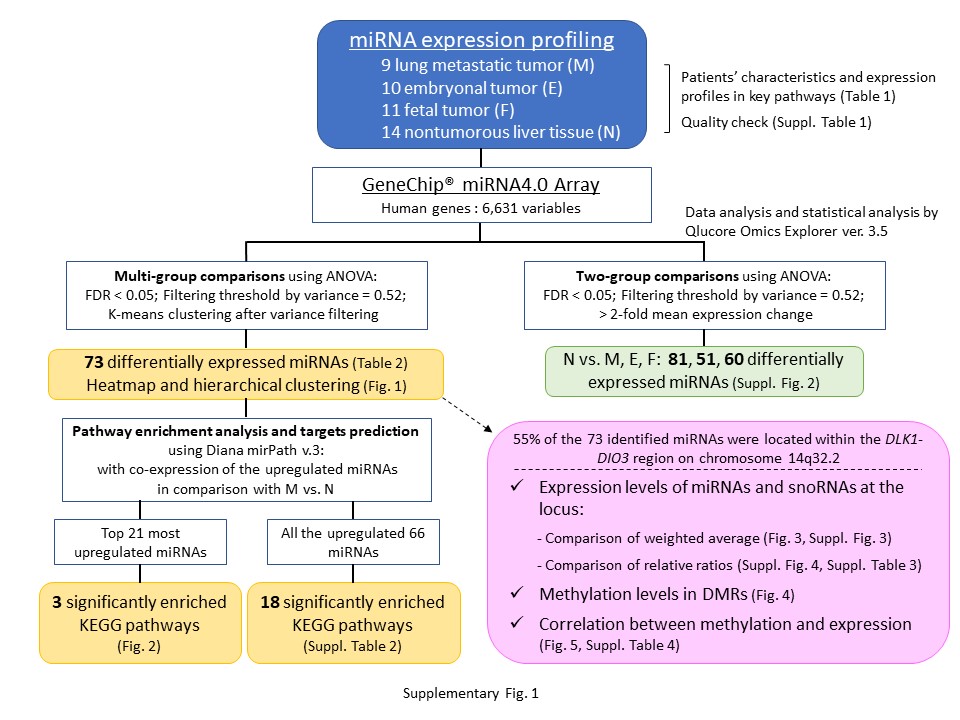

Supplement: Supplementary Figure 1 — Workflow describing the study design. [file Image_1.jpeg]

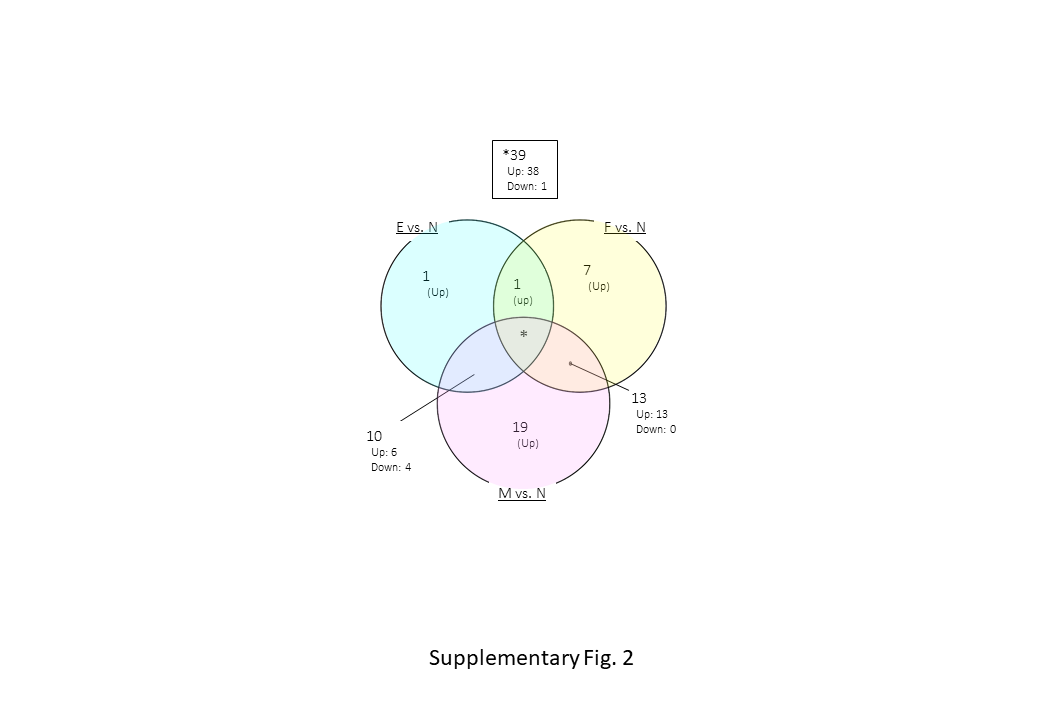

Supplement: Supplementary Figure 2 — Venn diagram showing the relationship between miRNAs which were up-/down-regulated in different HB stages of tissue and cell types compared to nontumorous surrounding liver. Each stage and cell type is illustrated as N, nontumorous surrounding liver; F, fetal subtype; E, embryonal subtype; M, metastatic tumor. [file Image_2.tiff]

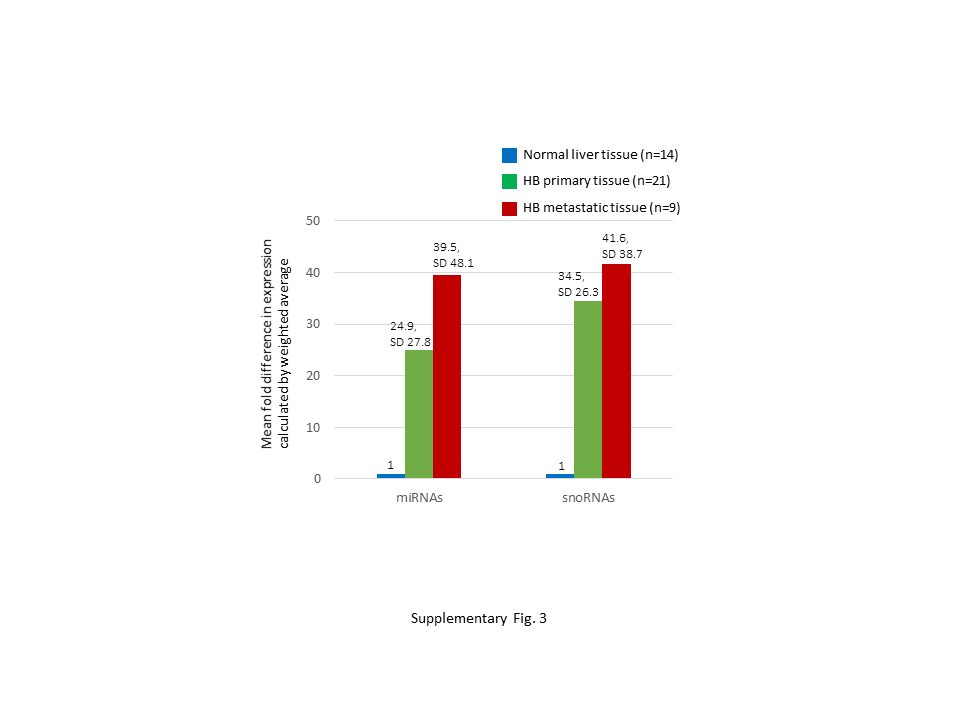

Supplement: Supplementary Figure 3 — Differential expression of miRNAs and snoRNAs at the 14q32.2 imprinted locus in HB metastatic and primary tissues versus normal liver tissues. [file Image_3.tiff]

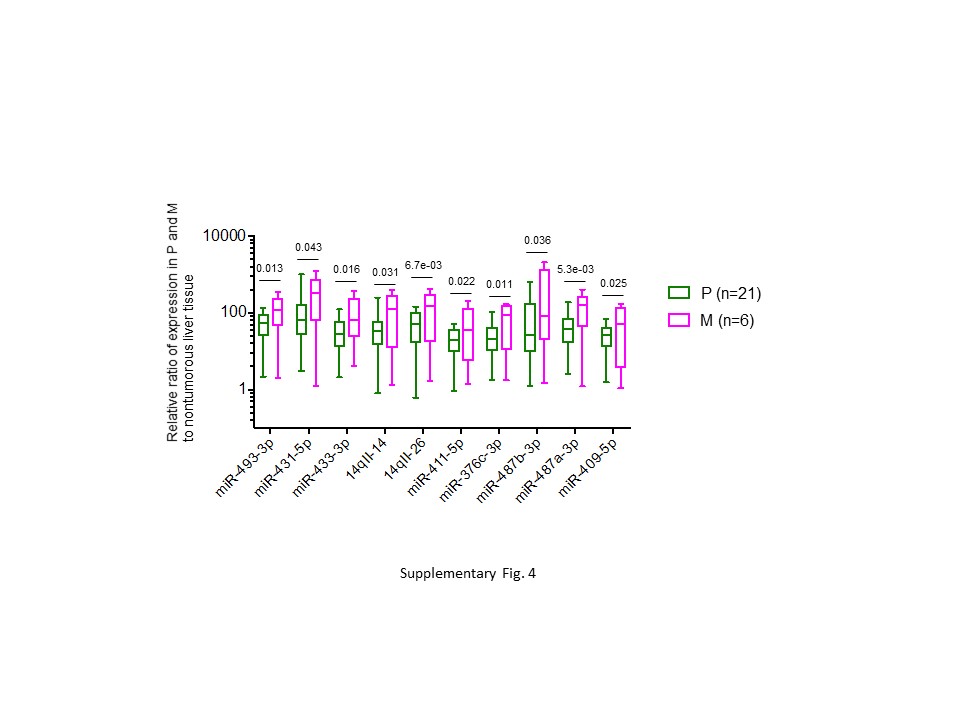

Supplement: Supplementary Figure 4 — Comparison of the expressions of 14q32 small RNAs which showed significant difference in primary tumors (n=21) vs. metastatic tumors (n=6). In this analysis, three metastatic tumors (21M, 23M-1 and 23M-2) were excluded because there were no matched normal liver tissues to calculate the relative ratio. Ten of the 31 small RNAs shown in Supplementary Table 3 are in this figure as representatives. The Y-axis shows the relative ratio of (Signal intensity in tumor/Signal intensity in the matched normal sample). The number above the boxes represents p-values calculated by Tukey’s test. The illustrative box plots present the median by a line in the box with the 10th, 90th percentile and the range of the relative ratios. P, primary tumors; M, metastatic tumors. [file Image_4.jpeg]

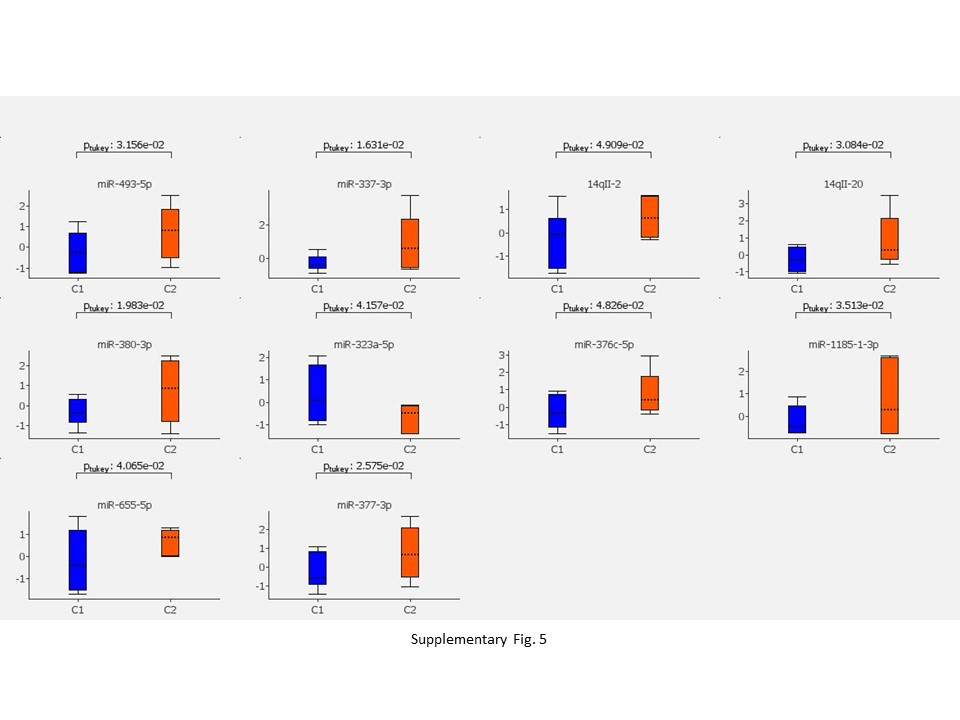

Supplement: Supplementary Figure 5 — Comparison of the expression level of 14q32 miRNAs and snoRNAs which showed significant differences in C2 (n=6) versus C1 tumors (n=15). The Y-axis shows the relative ratio of expression levels (signal intensity in tumor/signal intensity in the matched normal sample). The number above the boxes represents the p-values calculated by Tukey’s test. The box and whisker plots indicate the median as a line in the box, which demarcates the 10th, 90th percentiles of the range of the relative ratios. [file Image_5.tiff]

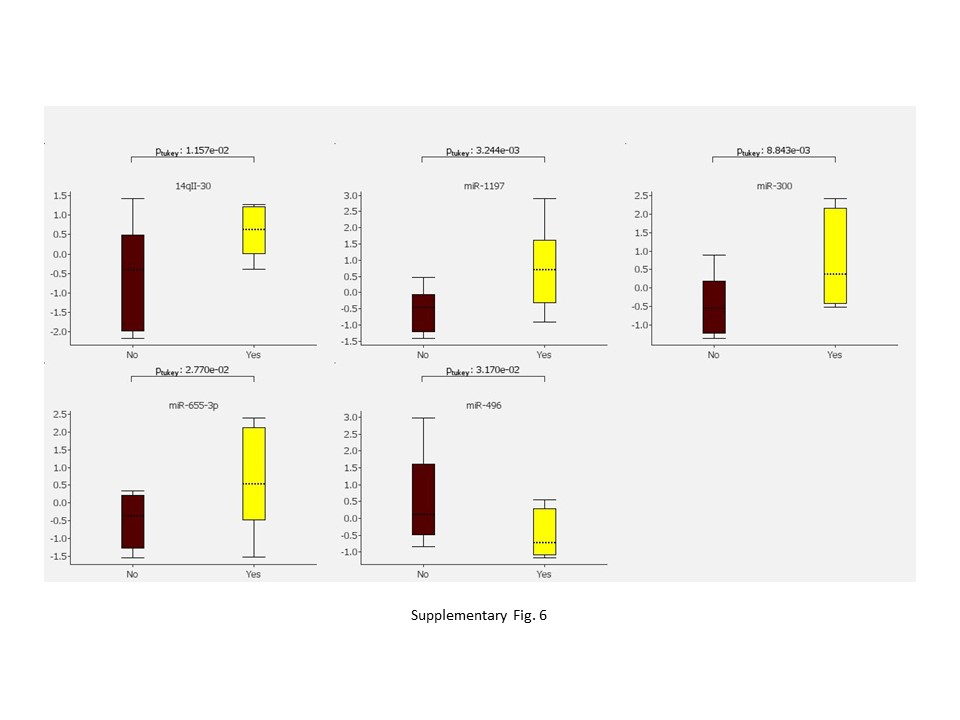

Supplement: Supplementary Figure 6 — Comparison of the expression level of 14q32 miRNAs and snoRNAs which showed significant difference in tumors exhibiting metachronous or synchronous metastasis (n=7) versus tumors that did not (n=14). The Y-axis shows the relative ratio of expression levels (signal intensity in tumor/signal intensity in the matched normal sample). The number above the boxes represents the p-values calculated by Tukey’s test. The box and whisker plots indicate the median as a line in the box, which demarcates the 10th, 90th percentiles of the range of the relative ratios. [file Image_6.tiff]
