# Supplementary material for: A MicroRNA Cluster in the DLK1-DIO3 Imprinted Region on Chromosome 14q32.2 Is Dysregulated in Metastatic Hepatoblastomas
Source: Front Oncol. 2020 Nov 12;10:513601. doi: 10.3389/fonc.2020.513601 (PMC7689214; doi:10.3389/fonc.2020.513601)
Supplement: Supplementary file 7 [file DataSheet_1.pdf]

Supplementary Table 1. Quality check data for 44 samples

Case No.	Sample ID	QC data		
		**Concentration** (ng/μl)	**OD 260/280 ratio**	**RIN**
1	22N	83.7	1.78	2.6
	22F	83.2	1.87	1.5
2	24N	84.7	1.79	1.9
	24F	84.6	1.75	2
	24E	83.2	1.77	1.7
3	H11N	149.2	1.77	2.6
	H11F	156.8	1.59	2.4
4	7N	169.3	1.68	2.5
	7E	192.8	1.66	2.5
	7M	154.5	1.64	2.5
5	H12N	126.4	1.95	1.5
	H12F	185.8	1.67	2.4
	H12E	133.6	1.91	2.1
6	H16N	85.1	1.69	1.8
	H16F	83	1.9	2.5
	H16E	82.5	1.84	2
7	25F-1	92.8	1.78	3.2
	25N	86.8	1.76	2
	25F-2	88.3	1.69	2.1
8	8N	198.1	1.79	2.4
	8F	201.4	1.81	2.3
	8E	191.6	1.82	2.3
	8M	194.8	1.69	2.4
9	H09N	150.8	1.81	2.5
	H09F	152.2	1.68	2.5
	H09E	151.2	1.8	2.2
10	2N	152.9	1.68	2.6
	2M	172.5	1.69	2.1
11	H10N	154	1.76	2.3
	H10F	190.1	1.72	2.6
	H10E	152.4	1.87	2.1
12	6N	153.5	1.65	2.6

	6F	185.9	1.62	2.4
	6E	153.6	1.63	2.4
	6M	158.1	1.63	2.4
13	23M-1	88.7	1.76	1.3
	23M-2	83.9	1.68	2.1
14	3N	150.3	1.87	2.5
	3E	179	1.82	2.4
	3M	155.4	1.72	2.2
15	9N	152.4	1.65	2.6
	9E	151.9	1.58	2.5
	9M	163	1.78	2.4
16	21M	84.9	1.84	2.1

RIN, RNA integrity number
