# Supplementary material for: A MicroRNA Cluster in the DLK1-DIO3 Imprinted Region on Chromosome 14q32.2 Is Dysregulated in Metastatic Hepatoblastomas
Source: Front Oncol. 2020 Nov 12;10:513601. doi: 10.3389/fonc.2020.513601 (PMC7689214; doi:10.3389/fonc.2020.513601)
Supplement: Supplementary file 8 [file DataSheet_2.pdf]

Supplementary Table 2. Significantly enriched pathways detected in pathway enrichment analysis and targets prediction (Diana mirPath v.3 web-based computational tool) of the 66[*] upregulated miRNAs in metastatic tumors compared to those in normal surrounding liver.

**KEGG pathway**	**p-value**
**1.** Fatty acid biosynthesis (hsa00061)	<1e-325
**2.** Prion diseases (hsa05020)	<1e-325
**3.** ECM-receptor interaction (hsa04512)	<1e-325
**4.** Adherens junction (hsa04520)	1.893252e-11
**5.** Viral carcinogenesis (hsa05203)	2.014489e-11
**6.** Proteoglycans in cancer (hsa05205)	2.625722e-11
**7.** Hippo signaling pathway (hsa04390)	8.28115e-10
**8.** Cell cycle (hsa04110)	8.251494e-07
**9.** Lysine degradation (hsa00310)	2.515057e-06
**10.** Protein processing in endoplasmic reticulum	1.718417e-05
**11.** Hepatitis B (hsa05161)	3.304304e-05
**12.** p53 signaling pathway (hsa04115)	6.020478e-05
**13.** Fatty acid metabolism (hsa01212)	0.0001324588
**14.** Pathways in cancer (hsa05200)	0.0003046663
**15.** Chronic myeloid leukemia (hsa05220)	0.001297946
**16.** TGF-beta signaling pathway (hsa04350)	0.007290645
**17.** Glioma (hsa05214)	0.008409624
**18.** Thyroid hormone signaling pathway (hsa04919)	0.02322047

[*]Of the 73 miRNAs shown in Table 1, four miRNAs (miR-378d, miR-378a-5p, miR-146b-3p and miR-203a) were downregulated in tumors. Out of the rest 69 miRNAs, three miRNAs (miR-127-3p, miR-543 and miR-127-5p) were excluded because they were not recognized by Diana mirPath v.3 web-based computational tool.
