# Supplementary material for: A MicroRNA Cluster in the DLK1-DIO3 Imprinted Region on Chromosome 14q32.2 Is Dysregulated in Metastatic Hepatoblastomas
Source: Front Oncol. 2020 Nov 12;10:513601. doi: 10.3389/fonc.2020.513601 (PMC7689214; doi:10.3389/fonc.2020.513601)
Supplement: Supplementary file 9 [file DataSheet_3.pdf]

Supplementary Table 3. Upregulated miRNAs and snoRNAs at 14q32 locus in metastatic tumors compared to primary tumors.

Gene Name	Genomic position		Tukey	Relative ratio in expression			
	Start	End	*p*-val	M/N		P/N	
				Average	SD	Average	SD
miR-493-5p	101335412	101335433	0.0456	47.5	42.7	23.3	17.8
miR-493-3p	101335453	101335474	0.0132	141.7	125.9	60.1	38.6
miR-431-5p	101347363	101347383	0.0427	427.2	450.5	145.2	225.6
miR-433-3p	101348286	101348307	0.0159	121.0	137.2	39.3	33.4
miR-432-3p	101350881	101350901	0.0118	4.8	3.8	1.9	1.7
miR-136-3p	101351087	101351108	0.0411	1.4	0.4	1.2	0.2
14qI-6	101405893	101405966	0.0144	40.9	41.3	14.8	12.2
14qI-9	101411986	101412056	0.0360	26.2	20.6	13.9	8.6
14qII-9	101432366	101432436	0.0482	28.5	29.4	14.1	8.1
14qII-11	101434448	101434521	1.25E-04	3.3	1.7	1.3	0.6
14qII-13	101436216	101436288	0.0193	7.3	8.9	2.4	1.7
14qII-14	101438440	101438513	0.0311	154.4	161.1	56.5	65.1
14qII-20	101447341	101447411	0.0133	17.6	21.0	5.1	4.1
14qII-26	101453383	101453453	6.71E-03	167.9	159.7	56.1	44.3
miR-411-5p	101489677	101489697	0.0217	65.1	78.0	22.6	15.2
miR-411-3p	101489712	101489733	0.0415	22.4	22.9	9.9	8.2
miR-380-5p	101491358	101491379	0.0398	2.7	2.6	1.3	0.8
miR-758-3p	101492408	101492429	5.77E-03	39.3	34.8	14.2	10.0

miR-376c-3p	101506069	101506089	0.0108	84.7	75.5	30.7	28.7
miR-654-3p	101506606	101506627	0.0253	63.4	60.7	24.1	25.8
miR-381-5p	101512264	101512285	0.0285	5.3	3.4	2.9	1.9
miR-487b-3p	101512842	101512863	0.0355	560.7	851.4	130.7	192.7
miR-539-5p	101513666	101513687	0.0461	16.1	19.7	6.1	5.9
miR-889-5p	101514248	101514269	0.0423	1.5	0.8	1.1	0.3
miR-487a-3p	101518831	101518852	5.27E-03	168.6	144.5	55.8	52.2
miR-668-3p	101521637	101521659	0.0311	13.1	13.5	5.1	5.1
miR-485-5p	101521764	101521785	0.0322	18.3	15.5	19.8	13.8
miR-409-5p	101531651	101531673	0.0251	69.1	70.4	29.2	20.1
miR-369-5p	101531943	101531964	0.0194	7.8	6.6	3.3	2.7
miR-410-5p	101532262	101532282	0.0444	1.2	0.4	1.0	0.2
miR-410-3p	101532298	101532318	0.0192	33.0	16.1	12.7	11.6

Genomic positions are based on GRCh37/hg19 build.; Relative ratio in expression was calculated by (Signal intensity in tumor/ Signal intensity in the matched normal sample) comparing M (metastatic tumors), P (primary tumors) to N (nontumorous surrounding liver samples). *P*-values were calculated by Tukey’s test.
