# Supplementary material for: A MicroRNA Cluster in the DLK1-DIO3 Imprinted Region on Chromosome 14q32.2 Is Dysregulated in Metastatic Hepatoblastomas
Source: Front Oncol. 2020 Nov 12;10:513601. doi: 10.3389/fonc.2020.513601 (PMC7689214; doi:10.3389/fonc.2020.513601)
Supplement: Supplementary file 10 [file DataSheet_4.pdf]

Supplementary Table 4. Correlation between the methylation levels at the three probes and the expression levels of the highly expressed miRNAs and snoRNAs shown in Figure 3.

Gene Name	Genomic position		Fold change	Probe in Infinium HumanMethylation450 BeadChip					
	Start	End	(M/N)	cg10943497		cg12197579		cg13551098	
				Rank correlation coefficient	*p*	Rank correlation coefficient	*p*	Rank correlation coefficient	*p*
miR-493-3p	101335453	101335474	193.87	-0.678	0.0188	-0.685	0.0173	0.678	0.0188
miR-431-5p	101347363	101347383	256.99	-0.713	0.0121	-0.392	0.21	-0.531	0.0793
miR-433-3p	101348286	101348307	72.15	-0.734	0.00905	-0.657	0.024	-0.783	0.00412
miR-432-5p	101350833	101350855	118.7	-0.72	0.011	-0.566	0.059	-0.65	0.0259
14qI-4	101402828	101402901	46.96	-0.643	0.028	-0.455	0.14	-0.643	0.028
14qI-7	101407463	101407538	54.08	-0.699	0.0145	-0.594	0.0458	-0.671	0.0204
14qII-3	101419686	101419759	72.42	-0.706	0.0133	-0.524	0.0839	-0.678	0.0188
14qII-14	101438440	101438513	90.34	-0.692	0.0159	-0.538	0.0749	-0.699	0.0145
14qII-17	101441143	101441216	51.18	-0.671	0.0204	-0.58	0.0521	-0.573	0.0555
14qII-21	101448312	101448382	47.56	-0.72	0.011	-0.587	0.0488	-0.608	0.04
14qII-22	101449263	101449333	51.72	-0.692	0.0159	-0.573	0.0555	-0.664	0.0222
14qII-26	101453383	101453453	139.25	-0.559	0.0627	-0.364	0.246	-0.524	0.0839
14qII-28	101455467	101455537	53.71	-0.72	0.011	-0.587	0.0488	-0.608	0.04
miR-411-5p	101489677	101489697	61.03	-0.643	0.028	-0.406	0.193	-0.643	0.028
miR-376c-3p	101506069	101506089	47.96	-0.678	0.0188	-0.476	0.121	-0.636	0.0301
miR-376a-3p	101506455	101506475	69.29	-0.762	0.0059	-0.378	0.227	-0.413	0.184

miR-487b-3p	101512842	101512863	83.58	-0.699	0.0145	-0.566	0.059	-0.699	0.0145
miR-487a-3p	101518831	101518852	147.06	-0.636	0.0301	-0.413	0.184	-0.587	0.0488
miR-485-3p	101521801	101521822	59.39	-0.741	0.00817	-0.51	0.0936	-0.469	0.127
miR-409-5p	101531651	101531673	64.66	-0.692	0.0159	-0.455	0.14	-0.671	0.0204

Genomic positions are based on GRCh37/hg19 build.; Fold changes were calculated by weighted average comparing M (metastatic tumors) to nontumorous surrounding liver samples using Transcriptome Analysis Console ver. 4.0. Spearman correlation coefficient was used to assess the associations between the methylation levels and the expression levels.
